# *SLC6A3* (*DAT1*) as a Novel Candidate Biomarker Gene for Suicidal Behavior

**DOI:** 10.3390/genes12060861

**Published:** 2021-06-04

**Authors:** Ekaterina Rafikova, Maria Shadrina, Peter Slominsky, Alla Guekht, Alexey Ryskov, Dmitry Shibalev, Vasiliy Vasilyev

**Affiliations:** 1 Institute of Gene Biology Russian Academy of Sciences, 119334 Moscow, Russia; ryskov@mail.ru (A.R.); Dsh1978@rambler.ru (D.S.); shunka@mail.ru (V.V.); 2 Institute of Molecular Genetics of National Research Centre, Kurchatov Institute, 123182 Moscow, Russia; shadrina@img.ras.ru (M.S.); slomin@img.ras.ru (P.S.); 3 Moscow Research and Clinical Center for Neuropsychiatry of the Healthcare Department, 115419 Moscow, Russia; neurodzm@mail.ru

**Keywords:** *SLC6A3*, *DAT1*, *COMT*, *SLC6A4*, *5HTT*, suicidal behavior, depressive symptoms, anxiety, gene polymorphism

## Abstract

It has been previously shown that the serotonin and dopamine neurotransmitter systems might influence the predisposition to suicidal behavior. This study aims to estimate the contribution of 11 polymorphisms in the genes *SLC6A4* (*5HTT*), *HTR1A*, *HTR2A*, *HTR1B*, *SLC6A3* (*DAT1*), *DRD4*, *DRD2*, *COMT*, and *BDNF* to suicidal behavior and severity of symptoms of depression and anxiety in the Russian population. The study was performed on 100 patients with repeated suicide attempts and 154 controls. We first found an association between *SLC6A3* (*DAT1*) 40 bp VNTR locus and suicidal behavior. This association was significant; when using the codominant (*p* = 0.006), dominant (*p* = 0.001), overdominant (*p* = 0.004), and log-additive (*p* = 0.004) models, LL genotype played a protective role (OR = 0.48, 0.29–0.82, *p* = 0.005). Difference in the distribution of *COMT* rs4680 genotypes was significant in the codominant (*p* = 0.04), dominant (*p* = 0.013), and log-additive (*p* = 0.02) models, and AA genotype might protect against suicide (OR = 0.49, 0.26–0.91, *p* = 0.025). SLC6A4 5-HTTLPR + rs25531 locus was significant in the recessive model (*p* = 0.024), and also affected the severity of symptoms of depression (*p* = 0.044) and personal anxiety (*p* = 0.029). Our results suggest that allelic variants of *SLC6A3*, *COMT*, and *SLC6A4* genes might be considered as risk factors for suicidal attempts.

## 1. Introduction

According to the World Health Organization, more than 800,000 people die from suicide each year [1]. Many family, twin, and adoption studies provide evidence for familial transmission of suicide and suicidal behavior [2,3,4,5,6]. The contribution of genetic risk factors was confirmed even after controlling for hereditary mental disorders [7]. The aggregation of suicide in families also cannot be fully explained by a similar environment [8]. Molecular studies have shown that suicide attempts could be associated with altered serotonin and dopamine transmission [9,10,11,12]. Given the above data, the search for an association between suicidal behavior and genes encoding important pathways of serotoninergic and dopaminergic transmission is relevant.

The promoter region of the serotonin transporter gene (*SLC6A4*/5-*HTT*) contains the variable number tandem repeats (VNTR) polymorphism (5-HTTLPR). Short (S) allele with 14 repeats is associated with lower expression activity compared to long (L) allele with 16 repeats [13,14]. Later single nucleotide polymorphism (SNP) A → G (rs25531) was detected within the sixth repeat of the S- and L-alleles. As was shown, the expression level of the L_G_ allele is lower compared to the L_A_ allele [15]. A recent meta-analysis demonstrated a lack of association between low-expressing alleles or genotypes and suicidal behavior, but low-expressing alleles (S + L_G_) were associated with violent suicide attempt [16]. Polymorphism C(–1019)G (rs6295) exists in the promoter region of the *HTR1A* gene. It might enhance or decrease gene expression depending on the location on the presynaptic or postsynaptic membrane [17,18]. A meta-analysis failed to find any associations between *HTR1A* rs6295 locus and suicidal attempts [19]. Postmortem study has discovered that the *HTR1B* gene polymorphism G861C (rs6296) affects gene expression activity and, as a result, changes the density of the receptors [20]. A meta-analysis showed the lack of association between *HTR1B* rs6295 locus and suicidal attempts [21]. In the case of *HTR2A* gene polymorphism A1438G (rs6311), it was shown that the A-allele increases the promoter activity [22]. Meta-analysis detected evidence for a significant association between *HTR2A* rs6311 and suicidal behavior [23].

A 40 bp VNTR-polymorphism exists in the 3′-UTR region of the dopamine transporter gene (*SLC6A3/DAT1*). Several studies have confirmed the effect of the length of this locus on gene expression, although contradictory results have been obtained [24,25,26,27]. It was shown that the 9R-allele may result in a high risk of depression and angry–impulsive personality traits [28,29]. We do not know about studies of an association between this locus and attempted suicide. The dopamine receptor gene (*DRD4*) contains a 120 bp VNTR polymorphism in the promoter region and 48 bp VNTR-polymorphism in exon 3. *DRD4* 120 bp VNTR polymorphism might affect gene expression activity—the long allele is associated with a lower expression level than the short allele [30]. To our knowledge, an association of this locus with suicidal behavior was not studied. The influence of the *DRD4* 48 bp VNTR-polymorphism on receptor activity is not well understood. The 7-repeat variant of this locus was found to be associated with such personality traits as impulsivity, aggression, depression, and novelty-seeking behavior, but not with attempted suicide [31,32,33]. The density of the DRD2 receptors in the striatum depends on the allelic variant of the rs1800497 polymorphism. It was shown that T-allele carriers have a lower density of DRD2 receptors [34]. The DRD2 rs1800497 polymorphism is associated with impulsivity and suggested to be related to suicidal attempts [35,36].

The enzyme catechol-O-methyltransferase (COMT) is responsible for the breakdown of catecholamines, including dopamine, adrenaline, and norepinephrine [37]. A meta-analysis of six studies has demonstrated that the *COMT* rs4680 polymorphism has a modestly significant association with suicidal behavior [21]. A recent meta-analysis demonstrated a lack of association between *COMT* rs4680 polymorphism and suicidal behavior in the overall population, but this locus was shown to be a risk factor in Asian populations [38].

The *BDNF* gene encoding the brain-derived neurotrophic factor contains a common polymorphism rs6264 which leads to the replacement of methionine with valine (Val66Met). Met-allele is associated with decreased protein activity [39] and possibly with suicide attempts [40].

The studies mentioned above are conducted in European, Asian, or general populations. Since ethnicity plays an important role in genetic association studies, it is necessary to consider the results of studies conducted on Slavic populations. In the Croatian population, no significant association was found between 5-HTTLPR polymorphism and suicide attempts; however, the L-allele was more common among suicide victims and the L10 haplotype (allele L of the 5-HTTLPR locus and allele 10R of the intron 2 VNTR-polymorphism of *SLC6A4* gene) was a risk factor for suicide [41]. The study on the Slovenian population showed no association between serotonin transporter polymorphisms and suicide [42]. Genetic variants of serotonin receptors also were not associated with suicidal behavior in the Slovenian population [43,44]. The combined Met/Met and Met/Val genotypes of the *BDNF* Val66Met variant were shown to be the risk factor for violent suicide in female subjects and for suicide in victims exposed to childhood trauma in Slovenians [45]. The conflicting data were obtained on the association of *COMT* polymorphism with suicide in Slovenian and Croatian populations [46,47].

Research findings on genetic risk factors for suicidal behavior are inconsistent and some results depend on the ethnicity of the participants. This study aims to estimate the contribution of 11 polymorphisms in the genes *SLC6A4* (*5HTT*), *HTR1A*, *HTR2A*, *HTR1B*, *SLC6A3* (*DAT1*), *DRD4*, *DRD2*, *COMT*, and *BDNF* to suicidal behavior and severity of symptoms of depression and anxiety in the Russian population. As described above, all polymorphisms under study affect gene expression, protein product activity, or were shown to be associated with personality traits.

## 2. Materials and Methods

### 2.1. Subjects

This study was conducted on the East Slavic population of Central Russia. The genetic diversity of the populations of Russia, including the East Slavic populations, has been studied previously. Most SNPs and insertion–deletion polymorphisms are common to all Slavic populations [48]. To minimize genetic heterogeneity in our samples, we collected all samples in the same geographic region—in Moscow and regions of Central Russia. A sample of patients (*n* = 100) who had attempted suicide at least two times and were monitored by a psychotherapist was used for this study (20 men and 80 women; mean age: 31.54 ± 11.13 years). A survey of patients and blood samples was carried out at the Moscow Research and Clinical Center for Neuropsychiatry of the Healthcare Department of Moscow. Depressive symptoms were evaluated with the help of Hamilton’s Depression Rating Scale (HAMD) and Beck’s Depression Inventory (BDI). Spielberger’s test was used for evaluation of the degree of situational and personal anxiety. The patients were diagnosed with the following: depressive episode (F 32.1), *n* = 22; recurrent depressive disorder (F 33.1), *n* = 22; mixed anxiety and depressive disorder (F 41.2), *n* = 13; bipolar disorder (F 31.3), *n* = 10; schizopathic disorder (F 21.8), *n* = 6; emotionally unstable personality disorder (F 60.31), *n* = 5; other, *n* = 24. The inclusion criteria were as follows: East Slavic origin and repeated suicide attempts. The exclusion criteria were as follows: alcoholism and drug addiction in the patient’s life history; serious neurological diseases (stroke, Parkinson’s disease, dementia, epilepsy, etc.); and severe somatic diseases (oncology). Demographic and clinical characteristics of patients are shown in Table 1.

- Patients cohort included 80 females and 20 males, the table shows the values for the total sample, including females and males.
- BDI—Beck’s Depression Inventory.
- HAMD—Hamilton’s Depression Rating Scale.
- SA—Situational anxiety.
- PA—Personal anxiety.

The control group (*n* = 154) was a general population of the East Slavic population of the city of Moscow and the regions of Central Russia (59 men (38.3%) and 95 women (61.7%); mean age: 62.19 ± 9.45 years). The participants in the control group were not assessed for levels of anxiety and depression. There was a difference in age distribution between the two groups. The case group included patients of different ages, while the control group included people over the age of 40 to reduce the likelihood of the presence of a genetic predisposition to suicide, which has not yet manifested.

Since women predominate in our samples, we tested whether gender influences the distribution of genotypes of the studied loci. We excluded 40 women from the sample of patients so that the ratio of men to women was approximately the same as in the control group. Then, we compared the distribution of genotypes in this reduced sample (66.7% of women) and in our full sample of patients (80% of women) using Pearson chi-squared test. We found no significant differences: *SLC6A3* (40 bp VNTR), *p* = 0.86; *DRD2* (rs1800497), *p* = 0.87; *DRD4* (120 bp VNTR), *p* = 0.95; *DRD4* (48 bp VNTR), *p* = 0.55; *COMT* (rs4680), *p* = 0.3; *SLC6A4* (5-HTTLPR + rs25531), *p* = 0.64; *HTR1A* (rs6295), *p* = 0.84; *HTR2A* (rs6311), *p* = 0.69; *HTR1B* (rs6296), *p* = 0.86; *BDNF* (rs6264), *p* = 0.95. Then, we excluded 20 women from the control group, and compared the distribution of genotype frequencies in the reduced (55.9% of women) and full (61.7% of women) control samples: SLC6A3 (40 bp *VNTR*), *p* = 0.96; *DRD2* (rs1800497), *p* = 0.94; *DRD4* (120 bp VNTR), *p* = 0.85; DRD4 (48 bp *VNTR*), *p* = 0.98; *COMT* (rs4680), *p* = 0.94; *SLC6A4* (5-HTTLPR+rs25531), *p* = 0.95; *HTR1A* (rs6295), *p* = 0.89; *HTR2A* (rs6311), *p* = 0.92; *HTR1B* (rs6296), *p* = 0.68; *BDNF* (rs6264), *p* = 0.97. Thus, the change in the ratio of women and men did not affect the distribution of genotypes in our samples.

All blood samples were collected with the informed consent of the investigated persons after a participant’s personal statement signature. The Ethics Committee of the Institute of Molecular Genetics (Institute of Molecular Genetics, Russian Academy of Sciences, Kurchatov sq. 2, Moscow, Russia) approved the study (protocol 03\19, 19 February 2019). All blood samples and participants’ personal data were anonymized. Details of patients and controls characteristics and genotyping results are shown in Supplementary (Table S1).

### 2.2. DNA Isolation and Genotyping

Genomic DNA was obtained from 250 μL of EDTA-anticoagulated venous blood using innuPREP Blood DNA Mini Kit (Analytik Jena AG, Jena, Germany), according to the manufacturer’s recommendations. The SNP and VNTR genotyping were carried out using locus-specific PCR as described previously [49,50,51,52,53,54].

### 2.3. Statistical Analysis

Hardy–Weinberg equilibrium calculator software (https://wpcalc.com/en/equilibrium-hardy-weinberg/, accessed on 15 July 2020) was used to calculate the correspondence of the genotype distribution in the population sample to the Hardy–Weinberg equilibrium (HWE). A logistic regression approach was applied to establish associations between gene polymorphisms and risk of suicidal attempts. Negative binomial regression was used to analyze association between genotypes and count variables: severity of depressive symptoms (HAMD and BDI scales), personal anxiety, and situational anxiety. The statistical significance of polymorphisms was established with a likelihood-ratio test (LRT). The loci were excluded from the logistic and negative binomial regression models in order from highest to lowest *p*-value. Akaike information criterion (AIC) was used to choose the final model that best fit the data. All calculations were performed in the R statistical environment.

The following genetics models were tested:

- Codominant. This model assumes that each genotype can influence risk independently of the others.
- Dominant. Common allele homozygotes were tested against rare allele homo- and heterozygotes.
- Recessive. Rare allele homozygotes were tested against common allele homo- and heterozygotes.
- Overdominant. Heterozygotes were tested against both homozygotes.
- Log-additive. A trend test for the genotypes; according to this model, each allele changes the risk in an additive manner (i.e., the presence of two alleles doubles the risk compared to the presence of only one allele). The test was based on a logistic regression model and genotypes were coded as 0, 1, or 2, depending on the amount of minor alleles.

The strength of associations between allelic variants of studied polymorphic loci and suicidal behavior was estimated using odds ratios (ORs), with the corresponding 95% confidence intervals (95% CIs). All tests were conducted at a level of significance of *p* < 0.05.

Given the data suggesting the effect of length of *SLC6A3* 40 bp *VNTR* locus on gene expression and high activity of the 10R allele compared with the 9R allele, we classified cases and controls as carriers of the long (≥10) and short (<10) alleles. Because of the complexity of SLC6A4 organization, we analyzed the distribution frequencies of its allelic variants according to their functional characteristics. To verify whether alleles with low or high expression activity are associated with suicidal behavior, we grouped alleles and genotypes according to their expression levels: high (L_A_) and low (S and L_G_) alleles with high and low expression activity, respectively, and high/high (L_A_/L_A_), high/low (L_A_/S_A_, L_A_/L_G_), and low/low (S_A_/S_A_, S_A_/L_G_, L_G_/L_G_) genotypes. The alleles of the *DRD4* 48 bp VNTR were grouped into long (≥7) and short (<7) allelic variants.

## 3. Results

All polymorphisms were in the Hardy–Weinberg equilibrium in control and case samples. Table 2 represents the most statistically significant results of the logistic regression analysis.

We first found an association between *VNTR*-polymorphism of the *SLC6A3* (*DAT1*) gene and suicidal behavior. The difference in genotype distribution of this locus was significant in the codominant (*p* = 0.006), dominant (*p* = 0.001), overdominant (*p* = 0.004), and log-additive (*p* = 0.004) models. The LL genotype was more common in controls than in cases (69.3% vs. 52%) and was significantly associated with lower risk of suicide (OR = 0.48, 0.29–0.82, *p* = 0.005).

Another gene of the dopaminergic system associated with suicide attempts was the *COMT* gene. The effect of *COMT* rs4680 on the risk of suicidal attempts was significant in the codominant (*p* = 0.04), dominant (*p* = 0.013), and log-additive (*p* = 0.02) models. Genotype AA (Met/Met) prevailed in controls (29.4% vs. 17%) and protected against suicidal behavior (OR = 0.49, 0.26–0.91, *p* = 0.025). Genotype distribution of *SLC6A4* 5-HTTLPR + rs25531 polymorphism showed a significant difference only in the recessive model (*p* = 0.024). The low/low genotype protected against suicide (OR = 0.05, 0.26–0.96, *p* = 0.038). We found that the frequency of TT genotype of *BDNF* rs6264 was slightly higher in the cases compared to the control group (7% vs. 2.7%), while CC-genotype tends to be less common (61% vs. 70.7%). However, the difference in genotype distribution was not statistically significant.

No significant associations between suicidal behavior and *DRD2 rs1800497*, *DRD4* 120 bp *VNTR* and 48 bp *VNTR*, *HTR1A rs6295*, *HTR2A rs6311*, and *HTR1B rs6296* alleles and genotypes were discovered. All results of genotyping of cases and controls are shown in Supplementary Table S1.

**Figure 1:**
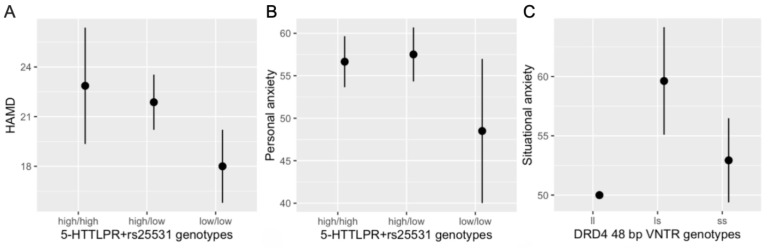
Levels of depression and anxiety in patients with suicidal behavior associated with studied loci. (**A**) Levels of depression according to HAMD and (**B**) levels of personal anxiety in patients with different genotypes of *SLC6A4* 5-HTTLPR+rs25531 locus; (**C**) levels of situational anxiety in patients with different genotypes of *DRD4* 48 bp VNTR locus.

To determine the effect of studied polymorphisms on the count variables, such as severity of depressive and anxiety symptoms, we conducted the negative binomial regression analysis. We observed a significant effect of *SLC6A4* 5-HTTLPR+rs25531 on depression symptoms (*p* = 0.044) and personal anxiety (*p* = 0.029) (Table 3). The low/low genotype prevailed in patients with lower levels of depression (HAMD) and personal anxiety (Figure 1A,B).

We also found significant associations between *DRD4* 48 bp VNTR-polymorphism and situational anxiety (*p* = 0.039). Carriers of SS genotype had lower levels of situational anxiety compared to carriers of LS genotype. Only one patient was homozygous LL, so the influence of this genotype is unclear (Figure 1C). Although *SLC6A3* (*DAT1*) and *COMT* genes were significant for the risk of suicidal attempts, they showed a lack of association with the severity of the symptoms of depression, situational anxiety, and personal anxiety.

## 4. Discussion

Genes related to neurotransmission and neurotrophic function have been widely studied in various mood and behavior disorders. To confirm possible associations studied previously and find out new associations, we investigated *SLC6A3* (40 bp VNTR), *DRD2* (rs1800497), *DRD4* (120 bp VNTR and 48 bp VNTR), *COMT* (rs4680), *SLC6A4* (5-HTTLPR + rs25531), *HTR1A* (rs6295), *HTR2A* (rs6311), *HTR1B* (rs6296), and *BDNF* (rs6264) polymorphic loci in patients with suicidal behavior. The study was carried out with a sample of patients of East Slavic origin from Central Russia who had attempted suicide at least two times. We supposed that the repeated suicidal attempts are not the result of impulsivity or mood disorders only, but show the genetic predisposition to suicidal behavior. We found statistically significant associations between suicidal behavior and *SLC6A3* (40 bp VNTR), *COMT* (rs4680), and *SLC6A4* (5-HTTLPR + rs25531) loci.

According to results of recent meta-analysis, *SLC6A4* 5-HTTLPR + rs25531 has no effect on suicidal behavior [16]. In the Russian cohort, this locus showed an association with suicide when using the recessive model. Homozygous genotype with low expression activity (low/low) played a protective role in our samples. Our data on the lack of association between *HTR1A* rs6295 and *HTR1B* rs6296 polymorphisms and suicide are consistent with previous meta-analyses [19,21]. A significant association between *HTR2A* rs6311 locus and suicide was shown in meta-analyses carried out in European and Asian populations [23], but we did not find this association in the Slavic cohort.

Although the association between *SLC6A3* 40 bp VNTR-polymorphism and mood disorders, as well as personality traits, was described [28,29], there was no information about an effect of this locus on suicidal tendencies. We discovered a strong association between the presence of short (<10 repeats) allele and suicidal behavior. The short allele (S) was more common in cases, while carriers of LL genotype had a lower risk of suicide. Given the data of low expression activity of the 9R-allele [27], we can suppose that suicidal behavior may be associated with dopamine transporter deficiency and, consequently, with increased dopamine signaling.

The effect of dopamine receptor genes polymorphism on suicidal behavior is not well understood. Although some studies have shown an association between *DRD2* rs1800497 and suicidal behavior [35,36], no differences between allelic and genotype frequencies of this locus in our samples of patients and controls were found. Similar to our research, previous studies failed to find an association between *DRD4* 48 bp VNTR-polymorphism and suicidal attempts [31,32]. We also did not find *DRD4* 120 bp VNTR locus to be associated with suicide. To our knowledge, this is the first study aiming to establish if the *DRD4* 120 bp VNTR polymorphism is related to suicidal behavior.

The AA (Met/Met) genotype of the *COMT* rs4680 locus played a protective role in the East Slavic cohort used in the present study. Our results are opposite to the results of the meta-analysis that demonstrated an association between Met (A) allele of *COMT* rs4680 and suicidal attempts. This study included Caucasian and Asian populations [21]. The latest meta-analysis showed lack of association between this locus and suicide in overall population, but there was a significant association in the Asian population [38]. These conflicting data indicate that the relationship between *COMT* polymorphism and suicidal behavior may differ depending on ethnicity.

In our samples, T(Met)-allele of *BDNF* rs6264 was more common in the cases, compared to the control group, which is consistent with a meta-analysis that showed the higher risk of suicide in carriers of the Met-allele [40]. However, the difference in genotypes distribution was not statistically significant in the present study.

It was previously shown that all of these genes might be associated with major depression, although conflicting results have been obtained. We tried to verify if these loci affect severity of depressive and anxiety symptoms in patients with suicidal behavior. We observed an effect of *SLC6A4* 5-HTTLPR + rs25531 locus on depressive and personal anxiety symptoms and an effect of *DRD4* 48 bp VNTR-polymorphism on situational anxiety. *SLC6A3* (*DAT1*) and *COMT* genes were associated with the risk of suicide but had no effect on the severity of symptoms of depression and anxiety. These results suggest that an effect of these loci on suicidal attempts is not explained by their effect on severity of the mood disorder.

Our results on serotoninergic genes are consistent with other studies conducted in Slavic populations. In the Croatian population, the L-allele of the 5-HTTLPR locus had a tendency to be more common among suicide victims [41]. The rs25531 (A/G) polymorphism was described later and is not included in this study. In our study, LA-allele is more common in cases, while alleles LG and S play a protective role. The influence of serotonin receptor genes on the risk of suicide was not found either in ours or in an earlier study of the Slavic population [43,44].

The Met/Met and Met/Val genotypes of the *BDNF* Val66Met variant were shown to be a possible risk factor for susceptibility to suicide in Slovenian population [45], and these genotypes were more common among suicide victims in our sample. The AA (Met/Met) genotype of the *COMT* rs4680 locus protected against suicide in our samples; the same association was shown in Slovenians, but only in the male group [46]. To our knowledge, this is the first study to investigate the effect of *SLC6A3* 40 bp VNTR, *DRD4* 48 bp VNTR, *DRD4* 120 bp VNTR, and *DRD2* rs1800497 loci on suicide risk in the East Slavic population.

## 5. Conclusions

In this study, we estimated the contribution of *SLC6A4* (*5HTT*), *HTR1A*, *HTR2A*, *HTR1B*, *SLC6A3* (*DAT1*), *DRD4*, *DRD2*, *COMT*, and *BDNF* genes to the suicidal behavior and severity of symptoms of depression and anxiety in the East-Slavic population of Russia. We found a significant association between *SLC6A3* (*DAT1*) 40 bp VNTR locus and suicidal behavior. We also observed a significant difference in the distribution of COMT (rs4680) and *SLC6A4* (5-HTTLPR + rs25531) genotypes between patients and controls in our samples. *SLC6A4* (5-HTTLPR + rs25531) locus influenced the severity of symptoms of depression and personal anxiety in patients. *DRD4* 48 bp VNTR-polymorphism was significantly associated with levels of personal anxiety in patients but not with the risk of attempted suicide. Our results suggest that allelic variants of *SLC6A3*, *COMT*, and *SLC6A4* genes might be considered as risk factors for suicidal attempts.

This study has several limitations. The sample of patients consisted of 100 people, and the control group consisted of 154 people, which is not enough to test the interactions between genes. This study is also limited by the diversity of diagnoses in a sample of suicidal patients. Although we investigated the effect of the studied loci on the severity of depression and anxiety symptoms in our suicidal patients, we did not adjust for the risks of suicide associated with patients’ diagnoses due to the limited sample size. Further studies considering more factors and conducted using larger samples, homogeneous in terms of ethnicity and diagnoses, are required to verify if these polymorphic loci are risk factors only for suicidal behavior or if they increase the risk of suicide via their effect on the predisposition to mental illness.

## Figures and Tables

**Table 1:** Demographic and clinical characteristics of the patients with suicidal behavior.

	Age, Years	BDI	HAMD	SA	PA
min	18	8	7	25	25
max	77	53	35	80	80
mean, SD	31.54 ± 11.13	28.85 ± 9.58	21.19 ± 5.22	55.52 ± 12.93	56.44 ± 10.05

**Table 2:** Association between genes of dopamine and serotonin systems and suicidal attempts.

Genotype	Control, *n* (%)	Suicide, *n* (%)	Logistic Regression, Genetic Model; LRT, P	ORs, *P*, 95% CI
*SLC6A3* (DAT1) 40 bp VNTR				
LL	113 (69.3)	52 (52)	codominant: 10.16, 0.006 * dominant: 10.54, 0.001 ** overdominant: 8.14, 0.004 ** log-additive: 8.05, 0.004 **	0.48, 0.005 *, 0.29–0.82
LS	46 (28.2)	44 (44)		1.998, 0.009 *, 1.19–3.37
SS	4 (2.5)	4 (4)		1.66, 0.483, 0.4–6.78
*COMT* rs4680				
AA	48 (29.4)	17 (17)	codominant: 6.42, 0.04 * dominant: 6.11, 0.013 * log-additive: 5.39, 0.02 *	0.49, 0.025 *, 0.26–0.91
GA	78 (47.9)	53 (53)		1.23, 0.418, 0.75–2.02
GG	37 (22.7)	30 (30)		1.46, 0.188, 0.83–2.56
*SLC6A4* 5-HTTLPR + rs25531				
high/high	49 (31.2)	35 (35)	recessive: 5.06, 0.024 *	1.19, 0.528, 0.7–2.02
high/low	67 (42.7)	50 (50)		1.34, 0.251, 0.81–2.22
low/low	41 (26.1)	15 (15)		0.5, 0.038, 0.26–0.96
*BDNF* rs6264				
CC	183 (70.7)	61 (61)	recessive: 2.82, 0.093+ log-additive: 2.86, 0.09+	0.65, 0.08 +, 0.4–1.05
CT	69 (26.6)	32 (32)		1.3, 0.312, 0.78–2.14
TT	7 (2.7)	7 (7)		2.71, 0.069 +, 0.93–7.93

The table shows the results of testing the final logistic regression models that were selected for each genetic model using the Akaike information criterion (AIC). Not all genetic models are shown for each locus, because loci were excluded when fitting the logistic regression model, if its exclusion improved the quality of the model. LRT—likelihood-ratio test. ORs—odds ratios; ORs were calculated for each genotype vs. two other genotypes and belong to genotypes in the table. CIs—confidence intervals. * *p* < 0.05. ** *p* < 0.005. + *p* < 0.1.

**Table 3:** Association between genes of dopamine and serotonin systems and severity of depressive, situational anxiety, and personal anxiety symptoms.

Scale	Gene	Polymorphism	LRT	*P*
HAMD	*SLC6A4* (*5HTT*)	5-HTTLPR + rs25531	6.29	0.044 *
Personal anxiety	*SLC6A4* (*5HTT*)	5-HTTLPR + rs25531	7.09	0.029 *
	*HTR1B*	rs6296	5.03	0.081 +
Situational anxiety	*DRD4*	48 bp VNTR	6.46	0.039 *
	*SLC6A4* (*5HTT*)	5-HTTLPR + rs25531	3.85	0.146
	*HTR1A*	rs6295	4.26	0.119

LRT—likelihood-ratio test, * *p* < 0.05, + *p* < 0.1.

## Data Availability

All data generated or analyzed during this study are included in this published article and its Supplementary Information files.

## Supplementary Materials

The following are available online at https://www.mdpi.com/article/10.3390/genes12060861/s1, Table S1: Demographic and clinical characteristics and results of genotyping of patients and control group.
